# Pharmacist-led medication therapy management improving medication adherence in a patient with type 2 diabetes: case report

**DOI:** 10.3389/fmed.2026.1910565

**Published:** 2026-08-21

**Authors:** Hong Jiang, Jing Liu, Yin Ying, Mingwan Zhang, Lu Zhao

**Affiliations:** 1 Department of Pharmacy, Tongde Hospital of Zhejiang Province, Hangzhou, Zhejiang, China; 2 Department of Geriatrics, Tongde Hospital of Zhejiang Province, Hangzhou, Zhejiang, China; 3 Editorial Office, Tongde Hospital of Zhejiang Province, Hangzhou, Zhejiang, China

**Keywords:** medication adherence, medication therapy management, pharmacist intervention, regimen simplification, type 2 diabetes mellitus

## Abstract

**Background:**

Poor medication adherence is a major challenge in healthcare for patients with type 2 diabetes mellitus (T2DM), particularly those with long disease duration, multiple comorbidities, and complex therapeutic regimens. This case report describes the application of pharmacist-led MTM (medication therapy management) in a patient with T2DM presenting complex therapeutic regimens and poor adherence.

**Patients and methods:**

A 76-year-old female with a 30-year history of T2DM complicated by diabetic peripheral neuropathy, hypertension, hyperlipidemia, hepatic steatosis, coronary atherosclerosis, and severe osteoporosis. The patient presented with poor glycemic control and severe medication nonadherence, which was characterized by frequent missed doses, irregular glucose monitoring, and forgotten weekly semaglutide injections. A clinical pharmacist conducted comprehensive MTM, including a full medication review, patient education, systematic medication and blood glucose monitoring, development of an optimized treatment plan in collaboration with the medical team, and ongoing follow-up.

**Results:**

After 3 months, glycemic control improved significantly, with fasting blood glucose at 7.6 mmol/L, postprandial glucose at 10.2 mmol/L, and glycated hemoglobin (HbA1c) at 7.3%. The Medication Adherence Report Scale-5 (MARS-5) score increased from 8 to 21, and no severe hypoglycemic events or other adverse drug reactions occurred during hospitalization or follow-up.

**Conclusion:**

Improvements in medication adherence and clinical outcomes were achieved following comprehensive pharmacist-led medication therapy management integrated with individualized clinical management in this case.

## Introduction

Diabetes mellitus has emerged as one of the most critical global healthcare challenges in the 21st century. With the progression of the disease, it can lead to chronic and acute complications that are potentially life-threatening conditions (1). Epidemiological data released by the International Diabetes Federation in 2021 indicated that 537 million adults were living with diabetes, a figure projected to reach 783 million by 2045. Type 2 diabetes mellitus (T2DM) is the predominant subtype, accounting for more than 90% of all cases (2). Consequently, T2DM imposes a substantial burden on healthcare systems worldwide. The effective management of T2DM depends not only on selecting appropriate treatment regimens but also on consistent, long-term patient adherence (3).

Medication adherence, according to the World Health Organization, is defined as the extent to which a patient’s behavior corresponds with agreed recommendations from a healthcare provider, and is widely recognized as a key determinant of successful treatment outcomes in diabetes care (4). Low adherence remains a major global healthcare challenge that is particularly dangerous for individuals living with chronic conditions. It is estimated that approximately 50% of patients do not adhere to prescribed recommendations, resulting in therapeutic failure, disease progression, decreased quality of life, and an increased risk of hospitalization or mortality (5). To improve adherence, reliable assessment tools are essential. The Medication Adherence Report Scale-5 (MARS-5) is a self-reported five-item tool designed to assess patients’ medication non-adherence behaviors. Assessment is conducted on a continuous scale: Each of the five items is rated on a 5-point Likert frequency scale (5 = never, 1 = always), and the total score is computed by summing item responses, where higher scores indicate better self-reported adherence. The MARS-5 has demonstrated robust reliability and validity for clinical screening and research in patients with hypertension, asthma, and diabetes (6).

**Figure 1:**
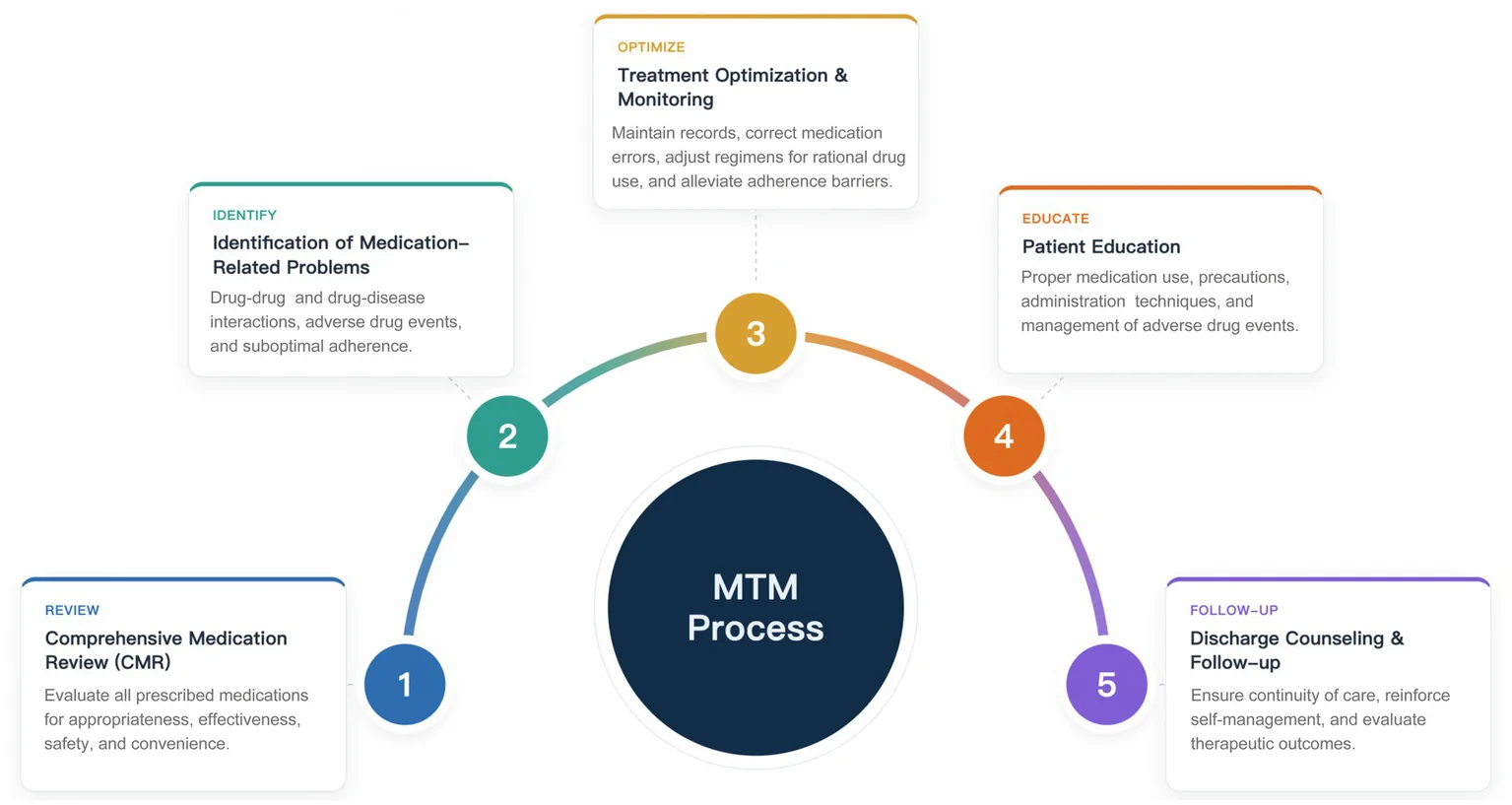
MTM process. MTM, medication therapy management.

High-quality, patient-centered healthcare rests on continuous monitoring and evaluation to ensure that all therapies are administered in an appropriate, safe, and effective manner. Pharmacist-led interventions encompass a variety of services. A primary responsibility of clinical pharmacists is to provide patient education and counseling on therapeutic strategies to optimize treatment outcomes, thereby supporting in the improvement or maintenance of patient health (7). Medication Therapy Management (MTM) originated in the United States to identify and resolve medication-related problems. MTM is a health service that is typically delivered by pharmacists to provide comprehensive, continuous, and integrated medication management services (8). Pharmacists, as medication therapy experts, play a pivotal role in helping patients optimize their medication regimens for these conditions through Medication Therapy Management services. They are responsible for identifying and resolving diverse medication-related problems, encompassing drug–drug and drug–disease interactions, and monitoring for necessary adjustments in cases of renal or hepatic impairment. The MTM process commences with a comprehensive medication review to ensure that all of the patient’s medication-related requirements are met and all prescribed agents are appropriate, effective, safe, and convenient. The whole process is implemented during the treatment. Throughout the course of therapy, pharmacists help patients maintain medication records, correct medication errors, and adjust therapeutic regimens to achieve optimal rational drug use, address barriers to adherence, and provide patient medication education regarding common adverse drug events (ADEs) and guidance on correct administration. In addition, discharge counseling with scheduled follow-up is provided to ensure continuity of care, reinforce self-management skills, and evaluate therapeutic outcomes. A personalized care plan is formulated and shared with the patient and the primary care provider to resolve and prevent medication-related problems. MTM also contributes to reducing healthcare expenditures for society and helps enhance the professional recognition of pharmacists (9). Currently, the value of MTM services has been widely demonstrated in clinical practice and constitutes a vital element of pharmaceutical care globally (Figure 1).

This case report describes the successful implementation of pharmacist-led MTM in an elderly patient with T2DM presenting with multiple comorbidities, and poor medication adherence.

## Case presentation

### Patient information

A 76-year-old woman (weight: 65 kg, height: 156 cm, body mass index: 26.7 kg/m^2^) was admitted to our hospital with a 30-year history of type 2 diabetes mellitus (T2DM) and poor glycemic control for preceding 1 month. The patient had been diagnosed with diabetic peripheral neuropathy (DPN), which was complicated by hypertension, hyperlipidemia, hepatic steatosis, coronary atherosclerosis, and severe osteoporosis.

On admission, the patient’s fasting blood glucose was 16.1 mmol/L, postprandial blood glucose was 21.5 mmol/L, and glycated hemoglobin (HbA1c) was 11.0%. The pre-admission antidiabetic regimen included acarbose 0.1 g three times daily, sitagliptin 100 mg once daily, insulin aspart 5 IU three times daily, insulin glargine 12 IU subcutaneously once daily, and semaglutide 0.25 mg weekly (used irregularly). The MARS-5 score was 8 of 25, reflecting significant non-adherence such as frequent missed doses, lack of routine glucose monitoring, forgotten injections of weekly semaglutide, and the inability to adhere to the complex four-times-daily insulin regimen.

### Therapeutic intervention

The clinical pharmacist participated in the multidisciplinary care team and delivered a comprehensive, continuous MTM intervention. The specific antidiabetic regimen is shown in Table 1.

**Table 1:** Antidiabetic regimen.

Time point	Antidiabetic regimen	Key pharmacist-led MTM actions
Day 1 (after admission)	Acarbose 0.1 g tid; Sitagliptin 100 mg qd; Insulin glargine 14 IU bedtime; Insulin aspart 5 IU tid; (Semaglutide 0.25 mg qw, 3 days prior)	Comprehensive medication review; treatment plan discussion; patient education; adherence evaluation
Day 3	Acarbose 0.1 g tid; Ertugliflozin 5 mg qd; Insulin glargine 16 IU bedtime; Insulin aspart 6 IU tid; (Semaglutide 0.25 mg qw, 5 days prior)	Treatment titration and monitoring
Day 5	Acarbose 0.1 g tid; Ertugliflozin 5 mg qd; IDegLira 16 U qd	Regimen simplification and monitoring; patient education
Day 8 (discharge)	Acarbose 0.1 g tid; Ertugliflozin 5 mg qd; IDegLira 20 U qd	Dose optimization; discharge planning and patient education
3-Month follow-up	Ertugliflozin 5 mg qd; IDegLira 20 U qd	Outcome assessment; patient education; adherence evaluation

Tid, three times a day; qd, once a day; qw, once a week; IDegLira, insulin degludec/liraglutide.

Day 1 (after admission): the patient presented with marked hyperglycemia. The clinical pharmacist developed an optimized glycemic regimen based on the patient’s individual clinical profile. A comprehensive assessment was conducted, which included gathering details, such as the duration of diabetes, previous treatment regimens, the degree of glycemic control, HbA1c levels, glycemic variability, and history of hypoglycemic events, and evaluation of the patient’s medical history. The patient was screened for the presence of acute or chronic diabetic complications. Furthermore, the pharmacist collected a complete medication history, encompassing prescription drugs, over-the-counter medications, and supplements, and assessed the patient’s adherence to these medications and any experienced adverse effects. Inquiries were also made regarding the patient’s lifestyle, including dietary and exercise habits. This information was used to select an appropriate pharmacological strategy tailored to the patient’s physiological status and disease condition. After discussion with the clinical team, a new intensive regimen was formulated, comprising four daily insulin injections (insulin glargine once daily plus insulin aspart three times daily) in combination with two oral antidiabetic agents, with the insulin glargine dose increased from 12 IU to 14 IU. Concurrently, targeted management for diabetic peripheral neuropathy, hypertension, hyperlipidemia, and osteoporosis was also initiated.

Day 2 to Day 4: the antidiabetic regimen was titrated based on point-of-care glucose monitoring. In response to clinical events during hospitalization, such as blood glucose fluctuations, worsening neuropathic symptoms, or potential adverse drug reactions, the pharmacist, referencing drug labeling, clinical guidelines, and the patient’s specific circumstances, collaborated with the team to adjust the drug type, dosage, or administration method, thereby ensuring both the efficacy and safety of treatment. Specifically, the patient’s glycemic control remained suboptimal. Based on the latest glucose readings, the pharmacist collaborated with the team to increase the doses of insulin glargine and insulin aspart. In addition, the pharmacist noted that the patient had coexisting coronary atherosclerosis and hypertension. In accordance with current guideline recommendations, and considering the proven cardiorenal protective effects of sodium-glucose cotransporter 2 (SGLT2) inhibitors in patients with type 2 diabetes and established cardiovascular disease (10), on Day 3, the pharmacist proposed switching from sitagliptin to once-daily ertugliflozin 5 mg. Before presenting this recommendation to the attending physician, the pharmacist comprehensively assessed potential drug–disease interactions and confirmed that there were no contraindications to SGLT2 inhibitor therapy. The proposal was subsequently accepted by the clinical team.

Day 5 to Day 7: the patient reported difficulty maintaining compliance with the multi-dose regimen, which included weekly subcutaneous semaglutide injections (the last dose having been administered 1 week earlier) in combination with four daily insulin injections. The patient acknowledged frequently forgetting the weekly semaglutide injection, and the cumulative injection burden was also identified as the principal barrier to adherence. Considering the core problem of the patient’s poor adherence, a regimen simplification was proposed by the clinical pharmacist. The complex multiple daily injections were transitioned to a fixed-ratio combination of insulin degludec and liraglutide (IDegLira) with only one injection per day. Because the weekly semaglutide injection was scheduled for Day 5, the switch to IDegLira was performed on that day to avoid missing the semaglutide dose and to prevent unnecessary duplication of glucagon-like peptide-1 receptor agonist (GLP-1RA) therapy. As liraglutide belongs to the same drug class as semaglutide, the pharmacist anticipated that this switch would preserve the incretin-based benefits of GLP‑1RA therapy previously provided by semaglutide while consolidating basal insulin and GLP-1RA delivery into a single injection, thereby substantially reducing the daily injection frequency. The dose was subsequently titrated based on the patient’s glycemic levels to optimize control. Concurrently, individualized medication monitoring and health education were provided, including guidance on correct injection technique and blood glucose monitoring, management strategies for adverse drug reactions, and key points of diabetes self-management.

At discharge (Day 8), the pharmacist delivered comprehensive medication counseling and scheduled a routine follow-up plan for the patient.

### Follow-up and outcomes

At the 3-month follow-up, glycemic control had improved significantly, with fasting blood glucose (FBG) at 7.6 mmol/L, postprandial glucose at 10.2 mmol/L, and glycated hemoglobin (HbA1c) at 7.3%. The MARS-5 score increased from 8 to 21, and no severe hypoglycemic events or other adverse drug reactions occurred during hospitalization or the follow-up period (Table 2). The patient reported a subjective reduction in injection-related distress and procedural anxiety following the pharmacist-led regimen simplification, which was attributed to the reduced injection frequency and was accompanied by structured patient education. These interventions collectively yielded a clinically meaningful enhancement in medication-taking behavior.

**Table 2:** Clinical outcomes: admission vs. 3-month follow-up.

Parameter	Admission	3-Month follow-up
Fasting blood glucose	16.1 mmol/L	7.6 mmol/L ✓
Postprandial blood glucose	21.5 mmol/L	10.2 mmol/L ✓
HbA1c	11.0%	7.3%✓
MARS-5 score	8	21 (better) ✓
Injection frequency	Four times daily + weekly	Once daily ✓

MARS-5, Medication Adherence Report Scale-5, higher scores reflecting better self-reported adherence; HbA1c, glycated hemoglobin.

## Discussion

This case report describes the case of a 76-year-old woman with long-standing type 2 diabetes mellitus (T2DM) complicated by multiple comorbidities and marked hyperglycemia, who achieved significant improvements in clinical outcomes and medication adherence. On admission, the patient presented with an MARS-5 score of 8 out of 25, indicating severe non-adherence characterized by frequent missed doses, irregular glucose monitoring, forgotten weekly semaglutide injections, and the inability to adhere to a four-times-daily insulin regimen. Through this multidisciplinary approach, the clinical pharmacist provided comprehensive MTM services, encompassing a full medication review, treatment plan discussion, adherence evaluation, treatment optimization and monitoring, outcome assessment, and structured patient education.

Poor medication adherence is a global healthcare challenge, particularly in patients with coexisting diabetes and other chronic diseases, where treatment complexity often leads to therapeutic failure (5). Our patient presented with at least six chronic conditions, requiring a polypharmacy regimen that included multiple daily injections (MDI) and various oral agents. The burden of managing multiple chronic conditions simultaneously often results in frequent missed doses, contributing to disease progression, elevated morbidity, and frequent hospital admissions (11). In this case, the patient’s inability to adhere to a four-times-daily insulin regimen and the irregular use of weekly semaglutide were the primary drivers of her uncontrolled hyperglycemia. Previous studies have consistently demonstrated that treatment complexity, including high injection frequency and the need for multiple administration times, is a strong predictor of poor adherence and persistence in patients receiving insulin therapy (12).

A pivotal step in this patient’s management was the simplification of the antidiabetic regimen. The transition from a complex MDI regimen and irregular GLP-1RA injections to a once-daily fixed-ratio combination of IDegLira addressed the patient’s primary barrier: the cumulative injection burden. In a 4-year real-world follow-up study, IDegLira demonstrated high effectiveness in poorly controlled patients, offering sustained glycemic control and high persistence rates that could be attributable to its simplified administration (13). Clinical trials have further demonstrated that IDegLira provides comparable or superior glycemic control with a lower risk of hypoglycemia and favorable weight benefits compared with basal-bolus insulin regimens (14). For patients facing significant adherence challenges, such simplified regimens represent a preferred therapeutic strategy. Notably, this regimen optimization was proposed by the clinical pharmacist during the MTM process.

The improvement in adherence, as reflected by the increase in the MARS-5 score from 8 to 21, was attributable not only to regimen simplification but also to the comprehensive educational and behavioral components of the MTM service. The clinical pharmacist delivered a structured MTM protocol encompassing a comprehensive medication review (CMR), identification of medication-related problems, treatment optimization and monitoring, patient education, and discharge counseling with structured follow-up. A meta-analysis of 40 randomized controlled trials evaluating pharmacist-led interventions in 8,822 patients demonstrated a statistically significant improvement in medication adherence among older adults (15). Similarly, a cluster randomized controlled trial involving 402 patients with concomitant type 2 diabetes mellitus and hypertension demonstrated that the adherence rate in the medication therapy management intervention group increased by 50% than the control group, underscoring that the multifaceted interventions exert a profound positive impact on medication adherence in patients with multiple cardiometabolic comorbidities (16).

The clinical pharmacist, as an integral member of the healthcare team, collaborated with the physicians to formulate a simplified and effective therapeutic plan while providing continuous patient education and follow-up. Beyond pharmacological optimization, personalized education and self-management support were pivotal to the observed clinical gains. Pharmacist-led education focusing on injection technique, blood glucose monitoring, prompt recognition of adverse effects, and lifestyle modification empowered the patient to actively engage in her care. Consistent with these observations, a meta-analysis demonstrated that pharmacist-led educational interventions significantly enhance self-care behaviors, medication adherence, and glycemic outcomes in patients with T2DM (17).

From the patient’s perspective, pharmacist-led interventions substantially enhanced medication adherence and disease self-management. Following the pharmacist-led simplification of the medication regimen and the consequent reduction in injection frequency, the patient reported a notable decline in injection-related distress and procedural anxiety. This reduction in overall treatment burden facilitated better integration of the therapeutic regimen into the patient’s daily routine, directly lowering the incidence of missed doses. The comprehensive individualized assessment conducted by the clinical pharmacist played a pivotal role in establishing therapeutic trust. The patient noted that this thorough evaluation ensured her specific clinical and personal circumstances were fully acknowledged and addressed, thereby fostering a robust sense of trust in the healthcare provider. Furthermore, targeted education provided by the clinical pharmacist effectively clarified the core pharmacotherapeutic goals for patients with diabetes. By establishing a clear understanding of dosing schedules and treatment rationale, the intervention successfully resolved her previous misconceptions. Consequently, the patient demonstrated enhanced self-efficacy in managing her condition autonomously.

While this case demonstrates a favorable outcome, it is subject to the inherent limitations of single-case reports and the absence of long-term follow-up data. The observed improvements may have been influenced by multiple confounding factors such as concurrent hospitalization, treatment intensification, regimen simplification, and pharmacist-led MTM—making it impossible to isolate and the independent contribution of any single component. Consequently, these, further findings require validation large-scale, well-designed prospective studies with extended observation periods to provide more robust evidence that MTM services can improve objective clinical outcomes and adherence in patients with diabetes. Furthermore, although besides, the MARS-5 is a validated adherence tool, it relies on patient self-report and may therefore be subject to recall bias or social desirability biases. In addition, objective clinical parameters such as body weight, BMI, blood pressure, renal function, and lipid profiles were not available at the 3-month follow-up because the patient only documented glycemic metrics, which limits the comprehensiveness of the clinical outcome assessment.

## Conclusion

In this case, pharmacist-led medication therapy management (MTM) was implemented throughout the entire course of care, providing safe, effective, rational, and personalized medication services for a patient with T2DM and severe medication non-adherence. This case demonstrated that the pharmacist-led MTM services in high-complexity patients are associated with marked improvements in glycemic control and medication adherence. However, these findings represent descriptive observations from a single case and cannot establish a causal link or the independent effectiveness of pharmacist-led MTM. Further rigorous, multi-center studies involving larger patient cohorts are required to validate these observations and evaluate long-term outcomes.

## Data Availability

The original contributions presented in the study are included in the article/supplementary material, further inquiries can be directed to the corresponding authors.
